# Heat-Related Mortality in a Warming Climate: Projections for 12 U.S. Cities

**DOI:** 10.3390/ijerph111111371

**Published:** 2014-10-31

**Authors:** Elisaveta P. Petkova, Daniel A. Bader, G. Brooke Anderson, Radley M. Horton, Kim Knowlton, Patrick L. Kinney

**Affiliations:** 1 National Center for Disaster Preparedness, Earth Institute, Columbia University, Suite 303, 215 W. 125th Street, New York, NY 10027, USA; 2 Center for Climate Systems Research, Columbia University, 2880 Broadway, New York, NY 10025, USA; E-Mails: dab2145@columbia.edu (D.A.B.); rh142@columbia.edu (R.M.H.); 3 Department of Environmental & Radiological Health Sciences, Colorado State University, 350 W. Lake Street, Fort Collins, CO 80523, USA; E-Mail: brooke.anderson@colostate.edu; 4 Department of Environmental Health Sciences, Mailman School of Public Health, Columbia University, 722 West 168th St., New York, NY 10032, USA; E-Mail: kknowlton@nrdc.org; 5 Natural Resources Defense Council, 40 W. 20th Street, New York, NY 10011, USA

**Keywords:** Heat-related mortality, climate change, heat impacts, United States, extreme temperatures

## Abstract

Heat is among the deadliest weather-related phenomena in the United States, and the number of heat-related deaths may increase under a changing climate, particularly in urban areas. Regional adaptation planning is unfortunately often limited by the lack of quantitative information on potential future health responses. This study presents an assessment of the future impacts of climate change on heat-related mortality in 12 cities using 16 global climate models, driven by two scenarios of greenhouse gas emissions. Although the magnitude of the projected heat effects was found to differ across time, cities, climate models and greenhouse pollution emissions scenarios, climate change was projected to result in increases in heat-related fatalities over time throughout the 21st century in all of the 12 cities included in this study. The increase was more substantial under the high emission pathway, highlighting the potential benefits to public health of reducing greenhouse gas emissions. Nearly 200,000 heat-related deaths are projected to occur in the 12 cities by the end of the century due to climate warming, over 22,000 of which could be avoided if we follow a low GHG emission pathway. The presented estimates can be of value to local decision makers and stakeholders interested in developing strategies to reduce these impacts and building climate change resilience.

## 1. Introduction

Heat is among the deadliest weather-related phenomena in the United States, killing more Americans in a typical year than floods, lightning and storms combined [1]. Global average temperatures have already increased by about 1.5 degrees F over the past 100 years [2] and could rise by an additional 2 degrees F by mid-century due to climate change [2,3]. As a result, the number of heat-related deaths may increase, particularly in urban areas where the mortality risk is exacerbated due to the high concentration of susceptible populations, as well as the enhancement of temperatures due to the urban heat island effect. Preparing for and preventing heat-related health problems is an increasing priority for public officials in cities across the country.

A growing number of studies have projected future heat-related mortality due to climate change in recent years [4,5,6,7,8,9,10,11,12,13,14,15], most of them predicting substantial increases. Projections of heat-related mortality may be of value to local stakeholders and decision makers as they start to consider the health impacts of climate change in long-term planning and policy. Regional adaptation planning is unfortunately often limited by the lack of quantitative information on potential future health responses.

The goal of the present analysis was to assess and report on future heat-related health risks in multiple US cities, potentially leading to improved understanding of weather and climate vulnerability in the health sector, and more informed risk management and adaptation decisions. Twelve northern US cities with populations over 250,000 were selected for evaluation because of their potential for vulnerability to future heat-related fatality risks under a changing climate. Downscaled temperature projections from 16 global climate models driven by two scenarios of greenhouse gas emissions were used to calculate heat-related premature fatalities in the 2020s, 2050s and 2080s. The presented estimates may be of value in developing strategies for reducing the future impacts of heat and building climate change resilience.

## 2. Data and Methods

**Figure 1:**
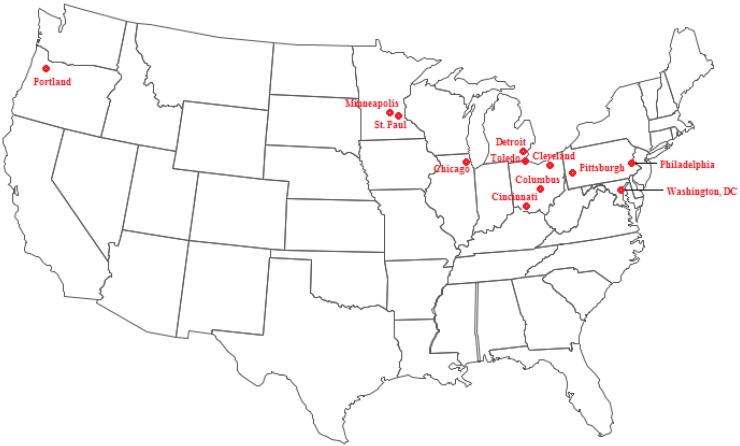
Cities analyzed in the present study.

Daily non-accidental mortality counts were obtained from an extended version of the National Morbidity, Mortality, and Air Pollution Study data set for 1987–2005 [16] for the 12 cities (mapped in Figure 1): Washington, DC, Chicago, IL, Detroit, MI, Minneapolis/St. Paul, MN, Cincinnati, OH, Cleveland, OH, Columbus, OH, Toledo, OH, Portland, OR, Philadelphia, PA, Pittsburgh, PA. Weather data were obtained from the National Climatic Data Center, Global Historical Climatology Network (NOAA NCDC GHCND) [17].

First, non-linear exposure-response functions relating daily risk of mortality to mean temperatures were fit for each city. The functions were generated using the dlnm package in R [18]. The exposure variable was the average of temperatures on lag days 0 and 1:

log(*µ*_t_) = *α* + *f*(*T*) + *δD* + *f*(*H*) + *f*(*L*)

where:

*µ*_t_ Daily count of mortality in the community for all non-accidental causes
*α* Model intercept
*f*(*T*) The basis used to model lag 0, 1 temperature using a natural cubic spline, 3 degrees of freedom (df)
*δ* Vector of coefficients for day of week
*D* Day of week for day *t*
*f*(*H*) Function of mean dew point temperature on day *t* (adjusted for daily mean temperature), modeled as a natural cubic spline, 3 degrees of freedom (df)
*f*(*L*) Function of time, modeled as a natural cubic spline with 7 df/year (used to model long-term and seasonal trends)

Next, temperature projections for each of the 12 cities noted above were obtained from 16 climate models and 2 Intergovernmental Panel on Climate Change (IPCC) greenhouse gas emissions scenarios (A2 and B1). The IPCC Special Report on Emissions Scenarios (SRES) represent a range of possible future pathways of carbon dioxide and other greenhouse pollutant emissions [19]. The A2 emissions scenario features high greenhouse gas concentrations by the end of the 21st century, with emissions growing throughout the entire century [20]. In contrast, the B1 emissions scenario includes societal changes that first reduces greenhouse gas emissions growth and before mid-century lead to emissions reductions, resulting in stabilization of greenhouse gas concentrations. Here the high (A2) and a low (B1) emission scenarios estimate an upper and lower bound for future heat-related mortality in the twelve cities. The global climate models are summarized in Table 1 below.

**Table 1:** Global-scale climate models for which downscaled data were used in the present study.

Climate Model Acronym	Institution	GCM Resolution (Degrees Latitude × Longitude)
BCCR	Bjerknes Center for Climate Research	1.9 × 1.9
CCCMA	Canadian Center for Climate Modeling and Analysis , Canada	2.8 × 2.8
CNRM	National Weather Research Center, METEO-FRANCE, France	2.8 × 2.8
CSIRO	CSIRO Atmospheric Research, Australia	1.9 × 1.9
GFDL1 (CM2.0)	Geophysical Fluid Dynamics Laboratory, USA	2.0 × 2.5
GFDL2 (CM2.1)	Geophysical Fluid Dynamics Laboratory, USA	2.0 × 2.5
GISS	NASA Goddard Institute for Space Studies	4.0 × 5.0
INMCM	Institute for Numerical Mathematics, Russia	4.0 × 5.0
IPSL	Pierre Simon Laplace Institute, France	2.5 × 3.75
MIROC	Frontier Research Center for Global Change, Japan	2.8 × 2.8
MIUB	Meteorological Institute of the University of Bonn, Germany	3.75 × 3.75
MPI	Max Planck Institute for Meteorology, Germany	1.9 × 1.9
MRI	Meteorological Research Institute, Japan	2.8 × 2.8
NCAR CCSM	National Center for Atmospheric Research, USA	1.4 × 1.4
NCAR PCM	National Center for Atmospheric Research, USA	2.8 × 2.8
UKMO	Hadley Center for Climate Prediction, Met Office, UK	2.5 × 3.75

Model projections were obtained for three future time slices, the 2020s, 2050s, and 2080s, as well as the baseline period of the 1980s as described in detail previously [21]. The modeled/future temperature data were derived from the Bias Corrected Spatial Disaggregated (BCSD) data set at 1/8 degree spatial resolution of Maurer *et al*. [22] and the observed National Oceanic and Atmospheric Administration, National Climatic Data Center, Global Historical Climatology Network (NOAA NCDC GHCND) historical data [17] was used for the base period (1970–1999). Each time slice represents a 30-year average of model outputs (e.g., the 2050s is actually 2040 to 2069), in order to minimize the influence of unpredictable interannual and decadal variability.

To calculate climate change-related increases in future temperatures in each city, the average of each calendar month’s 30 monthly values (using the BCSD dataset) for the future time periods were compared to the model results for the 1980s (1970–1999) baseline period. Mean temperature change projections are calculated as the difference between each model’s future simulation and the same model’s baseline simulation. Monthly changes through time from each of the 16 GCMs and two emissions scenarios were then applied to the observed daily station data from the 1980s to generate 32 time series of projected future “daily data”. This is a somewhat simplified approach to projections of extreme events, since it does not allow for possible changes in the patterns of climate variability through time. However, because changes in variability for most climate hazards are considered highly uncertain, the approach provides an initial evaluation of how extreme events may change in the future [21,23].

After deriving city-specific exposure-response functions and temperature projections, we calculated the daily relative risks for the 2020s, 2050s and 2080s by applying the city-specific exposure-response functions to modeled temperatures (from the 16 models and two scenarios) for those decades. Mortality was computed only on days when mean temperature was 60 °F (15.6 °C) or above, consistent with the modeling approach used previously [16].

We calculated heat-related daily deaths for both the baseline and the three future periods in each city, using a formula adapted from Annenberg and colleagues [24]:

$$\Delta Mortality=Y_{0}\times (\frac{RR-1}{RR})\times POP$$

where:

ΔMortality Daily heat-related additional premature deaths
*Y_0_*  Daily mortality rate (per 100,000 population)
*POP*  City population (divided by 100,000)
*RR*  City-specific exposure-response function

To focus on and isolate the climate effect, we kept other key inputs to the calculation constant in future decades. For example, we assumed constant population and mortality rates for the baseline and future periods. Population data for the thirteen cities was obtained from the 2010 U.S. Census (USDOC) [25]. The constant population assumption is a conservative one since many cities are projected to grow in the future, and have greater numbers of elderly, vulnerable people. Since city-specific mortality rates are not easily available for all cities, we used county-level mortality rates for the calculation. Future mortality rates could change due to changing patterns of obesity, heart disease or other factors. Counts of the numbers of deaths from all causes, and among all age groups, for 2010 for residents of each of the twelve counties in which the cities are located was obtained from the Centers for Disease Control and Prevention’s WONDER database (USCDC) [26].

Finally, for each city, we calculated the total number of heat-related deaths for the 30 year period centered on the 1980s using observed temperatures, and for the 2020s, 2050s, 2080s based on each of the sixteen climate models and the A2 and B1 greenhouse gas emissions scenarios. We also computed the relative percent change in deaths from baseline to each of the future time periods, attributable to the increase in daily heat under a changing climate.

## 3. Results

City populations and county-specific mortality rates are presented in Table 2.

**Table 2:** City populations and county-specific mortality rates.

City	State	County	City Population (2010)	County Population (2010)	County-Specific Crude Mortality Rate per 100,000 (2010)
**Chicago**	IL	Cook County	2,707,120	5,194,675	747.0
**Cincinnati**	OH	Hamilton County	296,943	802,374	939.3
**Cleveland**	OH	Cuyahoga County	396,815	1,280,122	1046.0
**Columbus**	OH	Franklin County	787,033	1,163,414	733.0
**Detroit**	MI	Wayne County	713,777	1,820,584	986.3
**Minneapolis**	MN	Hennepin County	387,753	1,152,425	673.6
**Philadelphia**	PA	Philadelphia County	1,526,006	1,526,006	920.6
**Pittsburgh**	PA	Allegheny County	305,704	1,223,348	1101.8
**Portland**	OR	Multnomah County	593,820	735,334	712.3
**St. Paul**	MN	Ramsey County	288,448	508,640	752.8
**Toledo**	OH	Lucas County	287,208	441,815	947.5
**Washington**	DC	District of Columbia	601,723	601,723	776.4

Projected warming is similar across the cities (the one exception is Portland, Oregon, which due in large part to the climatic influence of the Pacific Ocean, is projected to experience approximately 1 °C less warming than the other cities by the 2080s), especially in the 2020s and 2050s. The median projected warming (relative to the baseline) is about 1 °C by the 2020s, 2 °C by the 2050s, and 3.5 °C by the 2080s. However, by the 2080s there is broad spread across the models, ranging from a best case scenario of around 2 °C of warming to a worst case scenario of around 4.5 °C.

Summaries of annual heat-related deaths under a changing climate are presented for each city in Table 3. The first column lists the annual number of deaths during the baseline period, the 1980s. For the baseline period, city-specific exposure-response functions were used along with the historical weather data to calculate heat-related deaths. For each future decade, the 2020s, 2050s, and 2080s, respectively, the number of median heat-related deaths is calculated across the 16 global climate models (GCMs) and reported separately for the low emissions (B1) and high emissions (A2) scenarios. In addition, the annual number of excess heat-related deaths above the 1980s baseline is presented for each scenario during the three future time periods. For instance, there were 113 heat-related deaths in Washington, DC annually during the 1980s baseline period. During the 2020s, according to the B1 scenario, there would be around 136 heat-related deaths, or 23 deaths in excess of the 1980s baseline, while according to the A2 scenario, there would be 138 heat-related deaths or 25 deaths in excess of the 1980s baseline. By the 2080s, according to the B1 scenario, there would be around 163 heat-related deaths, or 49 deaths in excess of the 1980s baseline, while according to the A2 scenario, there would be 207 heat-related deaths, or 93 deaths in excess of the 1980s baseline. The central estimate of expected heat-related deaths in each city was rounded to the nearest whole number.

**Table 3:** Median number of expected heat-related annual deaths in the 2020s, 2050s and 2080s, and excess heat-related premature deaths compared to the 1980s. Median number of expected heat-related annual deaths is calculated across the 16 global climate models (GCMs) and shown separately for the low emissions (B1) and high emissions (A2) scenarios.

City	1980s	2010-2039 (2020s)				2040-2069(2050s)				2070-2099(2080s)			
		Low emissions Scenario		High emissions Scenario		Low emissions Scenario		High emissions Scenario		Low emissions Scenario		High emissions Scenario	
		(B1)		(A2)		(B1)		(A2)		(B1)		(A2)	
		Number of deaths	Deaths in excess of the 1980s baseline	Number of deaths	Deaths in excess of the 1980s baseline	Number of deaths	Deaths in excess of the 1980s baseline	Number of deaths	Deaths in excess of the 1980s baseline	Number of deaths	Deaths in excess of the 1980s baseline	Number of deaths	Deaths in excess of the 1980s baseline
Chicago	257	321	64	335	77	369	112	423	166	419	161	566	308
Cincinnati	14	17	3	17	3	19	4	21	6	20	6	25	11
Cleveland	41	53	11	55	13	60	19	69	28	68	27	93	51
Columbus	61	76	15	78	17	86	25	99	38	99	38	130	69
Detroit	116	148	32	152	36	168	52	185	69	187	71	250	134
Minneapolis	23	29	6	30	7	34	11	38	15	37	14	49	26
Philadelphia	278	334	56	345	67	375	97	416	138	415	137	526	248
Pittsburgh	26	33	7	34	8	37	12	43	17	43	17	56	30
Portland	62	80	18	81	19	92	30	103	41	104	42	142	80
St. Paul	19	24	5	25	6	28	9	32	13	31	11	41	22
Toledo	26	34	8	35	9	39	12	44	18	43	17	60	34
Washington	113	136	23	138	25	152	38	166	52	163	49	207	93

**Figure 2:**
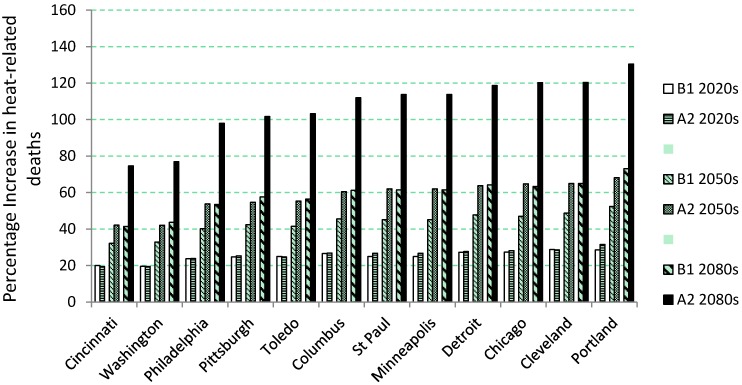
Percentage increase in heat-related deaths during 2020s, 2050s and 2080s compared to the 1980s baseline period for the twelve cities. Calculated across the 16 global climate models (GCMs) used in this study and displayed separately for the low emissions (B1) and high emissions (A2) scenarios.

Percent change in heat-related deaths for a typical year during the 2020s, 2050s and 2080s compared to the 1980s baseline for each of the twelve cities are presented graphically in Figure 2. Again, the percentage change is based on the number of median heat-related deaths calculated across the 16 global climate models (GCMs) and B1 and A2 scenarios. For instance, in Washington, DC, the average percentage increase in the number of deaths during the 2020s would be 19.5% according to the B1 scenario and 19.6% according to the A2 scenario. By the 2080s, an increase of 43.7% and 76.9% was projected for the same location according to the B1 and A2 scenarios, respectively.

All cities were projected to have substantial increases in heat-related fatalities in future decades, with increasing impacts over time, and for the A2 *vs*. B1 emissions scenario. Already by the 2020s, most cities showed increases in the 20% to 30% range. By the 2050s, deaths could increase by as much as 50% to 70% for some cities. Even higher impacts could be seen by the 2080s.

Cumulative heat-related deaths, computed by adding up the number of annual heat-related deaths which could result from each of the two emissions pathways, are presented in Table 4. Cumulative deaths may be a useful measure for decision makers for several reasons. First, calculating cumulative deaths over time provides a long term quantitative assessment of the impacts of heat. In Table 4, this has been done for 3 periods for each city, from the beginning of the baseline period to the end of each time slice, 1970–2039, 1970–2069 and 1970–2099, respectively. In Washington, DC, for example, 8757 cumulative heat-related deaths were calculated to occur until 2039 under the B1 and 8861 under the A2 scenario. By 2099, the number of cumulative heat-related deaths expected in Washington, DC, according to the B1 and A2 scenarios, was 18,184 and 20,050, respectively. The second useful application of cumulative deaths is in estimating the extra burden of mortality due to the higher emission scenario, A2 *vs*. the lower emissions scenario, B1. This estimate is provided in the last column of Table 4. For Washington, DC alone, the A2 emissions pathway was estimated to result in 1866 extra deaths by the end of the century.

**Table 4:** Cumulative median heat-related premature deaths by the end of each future decade, 1970–2039, 1970–2069 and 1970–2099. Calculated as a sum of annual median heat-related deaths since the beginning of the baseline period. Annual heat-related deaths between 2000 and 2009 were interpolated.

City	Cumulative Deaths						Extra Deaths from High Emissions Scenario Relative to Low Emissions Scenario (1970–2099)
	1970 to 2039		1970 to 2069		1970 to 2099		
	Low Emissions Scenario (B1)	High Emissions Scenario (A2)	Low Emissions Scenario (B1)	High Emissions Scenario (A2)	Low Emissions Scenario (B1)	High Emissions Scenario (A2)	
Chicago	20,334	20,869	31,409	33,565	43,971	50,530	6559
Cincinnati	1098	1117	1664	1742	2277	2507	229
Cleveland	3309	3388	5110	5457	7157	8238	1081
Columbus	4792	4894	7384	7858	10,358	11,768	1410
Detroit	9271	9438	14,325	14,980	19,939	22,490	2551
Minneapolis	1826	1857	2835	2999	3937	4474	537
Philadelphia	21,464	21,914	32,706	34,389	45,144	50,164	5021
Pittsburgh	2063	2100	3187	3384	4466	5060	594
Portland	4968	5018	7725	8110	10,854	12,374	1520
St. Paul	1518	1544	2357	2493	3273	3719	446
Toledo	2097	2144	3252	3476	4556	5274	718
Washington	8757	8861	13,304	13,840	18,184	20,050	1866

## 4. Discussion and Conclusions

We carried out an assessment of the potential impacts of climate change on heat-related mortality in 12 cities using 16 global climate models, driven by two scenarios of greenhouse gas emissions. The presented estimates can be of value to local decision makers and stakeholders interested in developing strategies to reduce these impacts and building climate change resilience.

Our analyses indicate that climate change may result in substantial increases in heat-related fatalities in all of the twelve cities included in this study. The magnitude of the projected increase differed across time, cities, climate models and greenhouse pollution emissions scenarios. In general, climate change was projected to result in increasing heat-related deaths over time throughout the 21st century, and more so under the high emission pathway. Our findings also highlight the potential public health benefits that could result from the lower greenhouse gas emissions scenario pathway at the city level. By the end of the century, the potential number of excess deaths due to the higher greenhouse gas concentration A2 scenario *versus* the reduced-emissions B1, calculated from the beginning of the baseline period, ranged from 131 in Portland, ME to 6559 in Chicago. There were a total of 22,553 estimated deaths across the 12 cities that could potentially be avoided by reducing greenhouse gas emissions.

Projecting future heat impacts on mortality required a range of assumptions. Factors such as city-specific temperature-mortality response functions, as well as underlying mortality rates and population assumptions all impact projections. In the present analysis, we isolated the climate change effect by holding these factors constant across time. This approach has important limitations. First, assuming that population in each city will remain constant at the 2010 Census level may lead to underestimation of future impacts in regions where urbanization continues throughout the century. Since population size is the main driver of inter-city differences in projected heat-related deaths, differences may get even bigger, and the absolute numbers larger, as urban populations increase. Mortality rates are also unlikely to remain constant in the coming decades if the average life-expectancy and the overall resilience of the population to temperature extremes continue to increase. On the other hand, social inequalities may persist and the proportion of elderly, vulnerable people, most susceptible to heat-related deaths is likely to increase in the future. Another limitation of the study is that we did not model population adaptation to heat, which has been documented in a number of studies [27,28,29,30,31] and which may result in our results overestimating mortality impacts. Also it is important to note that we did not account for cold-related mortality, which, by decreasing in the future, could balance some of the heat-related effects we projected. A recent study suggests that this could reduce slightly the future impact of climate-related warming [14]. Nonetheless, these assumptions allowed the estimation of potential heat-related mortality impacts due to climate change in each city, which can be of value in supporting local efforts to reduce heat-related vulnerability.

Despite the uncertainties in the change and interplay of the various population-level variables, future greenhouse gas emissions will have a measurable influence on future heat-related mortality. For instance, although the number of excess heat-related deaths projected under the low-emission (B1) and high-emission (A2) scenarios will likely be comparable during the 2020s, nearly twice as many deaths may occur annually by the 2080s under the high-emission compared to the low-emission scenario. Therefore, in addition to more direct health benefits associated with improved air quality, reducing greenhouse gas emissions can have a substantial impact on reducing future heat-related mortality in all of the twelve cities.
